# Intra- and Peritumoral Radiomics Model Based on Early DCE-MRI for Preoperative Prediction of Molecular Subtypes in Invasive Ductal Breast Carcinoma: A Multitask Machine Learning Study

**DOI:** 10.3389/fonc.2022.905551

**Published:** 2022-06-24

**Authors:** Shuhai Zhang, Xiaolei Wang, Zhao Yang, Yun Zhu, Nannan Zhao, Yang Li, Jie He, Haitao Sun, Zongyu Xie

**Affiliations:** ^1^ Department of Radiology, The First Affiliated Hospital of Bengbu Medical College, Bengbu, China; ^2^ Department of Radiology, Sir Run Run Shaw Hospital, Zhejiang University School of Medicine, Hangzhou, China; ^3^ Department of Radiology, Zhongshan Hospital, Fudan University, Shanghai, China; ^4^ Shanghai Institute of Medical Imaging, Department of Cancer Center, Zhongshan Hospital, Fudan University, Shanghai, China

**Keywords:** breast cancer, magnetic resonance imaging, dynamic contrast-enhanced imaging, radiomics, molecular subtype

## Abstract

**Purpose:**

The aim of this study is to investigate radiomics features extracted from the optimal peritumoral region and the intratumoral area on the early phase of dynamic contrast-enhanced MRI (DCE-MRI) for predicting molecular subtypes of invasive ductal breast carcinoma (IDBC).

**Methods:**

A total of 422 IDBC patients with immunohistochemical and fluorescence *in situ* hybridization results from two hospitals (Center 1: 327 cases, Center 2: 95 cases) who underwent preoperative DCE-MRI were retrospectively enrolled. After image preprocessing, radiomic features were extracted from the intratumoral area and four peritumoral regions on DCE-MRI from two centers, and selected the optimal peritumoral region. Based on the intratumoral, peritumoral radiomics features, and clinical–radiological characteristics, five radiomics models were constructed through support vector machine (SVM) in multiple classification tasks related to molecular subtypes and visualized by nomogram. The performance of radiomics models was evaluated by receiver operating characteristic curves, confusion matrix, calibration curves, and decision curve analysis.

**Results:**

A 6-mm peritumoral size was defined the optimal peritumoral region in classification tasks of hormone receptor (HR)-positive vs others, triple-negative breast cancer (TNBC) vs others, and HR-positive vs human epidermal growth factor receptor 2 (HER2)-enriched vs TNBC, and 8 mm was applied in HER2-enriched vs others. The combined clinical–radiological and radiomics models in three binary classification tasks (HR-positive vs others, HER2-enriched vs others, TNBC vs others) obtained optimal performance with AUCs of 0.838, 0.848, and 0.930 in the training cohort, respectively; 0.827, 0.813, and 0.879 in the internal test cohort, respectively; and 0.791, 0.707, and 0.852 in the external test cohort, respectively.

**Conclusion:**

Radiomics features in the intratumoral and peritumoral regions of IDBC on DCE-MRI had a potential to predict the HR-positive, HER2-enriched, and TNBC molecular subtypes preoperatively.

## Introduction

Breast cancer is one of the most common malignant tumors, and it has been the leading cause of cancer death among women aged 20 to 59 years (1, 2). The invasive ductal breast carcinoma (IDBC), accounting for approximately 80% in all breast cancers, is the most common histological subtype of breast cancer (3, 4). As a highly heterogeneous malignant tumor, IDBC included four main molecular subtypes including Luminal A, Luminal B, epidermal growth factor receptor 2 (HER2)-enriched, and triple-negative breast cancer (TNBC) (5, 6). More importantly, molecular subtypes of IDBC are closely correlated with treatment strategies, therapeutic effects, and clinical outcomes (7–9). Currently, IDBC molecular subtyping mainly relied on pathological immunohistochemistry (IHC) and gene expression profiling (10). However, both IHC and gene expression profiling are time-consuming and depend on resection or biopsy specimens, in which an accurate diagnosis before surgery is difficult to make. (11) Therefore, there has been more attention on developing new and noninvasive strategies to preoperatively assess molecular subtypes for guiding clinical decisions. MRI is commonly used in clinic for preoperative evaluation of breast cancer. Dynamic contrast-enhanced MRI (DCE-MRI) has been reported to reflect more detailed biological information of tumor through analyzing tumorous hemodynamic features (12, 13). Several studies have reported that texture features derived from DCE-MRI were correlated with diverse biomarker levels, such as estrogen receptor (ER), progesterone receptor (PR), and HER2 (14, 15).

Radiomics enables subtle imaging feature extraction and quantification for exploring the underlying associations between the features and tumoral pathophysiology (16). Recently, several studies found that the radiomics features extracted from DCE-MRI are partially correlated with the tumoral heterogeneity or biological behavior, including molecular subtypes, Ki-67 expression, and therapeutic effect of neoadjuvant chemotherapy (15, 17, 18). However, most previous studies mainly focused on the intratumoral region while neglecting the areas surrounding the tumor containing the peritumoral information. The interactions between intratumoral cells and peritumoral elements influence tumor evolution and progression (19). For example, interactions between tumor cells and mesenchymal cells in the peritumoral area could induce the cytokine release and promote the tumor immunosuppressive microenvironment formation, leading to tumor progression (20). Peritumoral edema and angiogenesis are correlated with the malignant behavior of the tumor (21, 22). Several studies have explored the radiomics features in the peritumoral areas based on various image types, such as mammography, ultrasound, and MRI, and had preliminary results in tumor diagnosis, biological indicator prediction, and prognosis evaluation (23–25). Li et al. recently reported that the radiomics features in the intra-/peritumoral regions based on DCE-MRI are able to identify the HER2 and Ki-67 status in breast cancer (26). In Li’s study, the peritumoral region was set as 4 mm from the tumor boundary; however, the optimal peritumoral size for breast cancer has rarely been studied.

In this study, we first aimed to clarify the optimal peritumoral region in differentiating the IDBC molecular subtypes. Moreover, the radiomics features in the intratumoral and peritumoral areas based on DCE-MRI were generated, and it was verified whether radiomics analysis in DCE-MRI is helpful for the preoperative discrimination of IDBC molecular subtypes.

## Methods and Materials

### Patient Population

This study was approved by the institutional review boards from both participating hospitals, and the requirement for informed consent was waived. The data of 472 IDBC women from the First Affiliated Hospital of Bengbu Medical College (Center 1, from May 2016 to March 2021) and 141 IDBC women from Sir Run Run Shaw Hospital, Zhejiang University (Center 2, from April 2018 to August 2019) were retrospectively analyzed. The patient inclusion criteria were as follows: 1) underwent DCE-MRI scans less than 1 month before biopsy or resection; 2) no surgery or treatment before MRI scans; 3) all patients were pathologically diagnosed by surgical resection sample or needle biopsy; and 4) solitary tumor. Patient exclusion criteria were as follows: 1) more than a month between histopathological examination and MRI scans; 2) tumor size less than 1 cm; 3) patients with incomplete clinical, imaging, and pathological data; 4) inadequate image quality.

**Figure 1:**
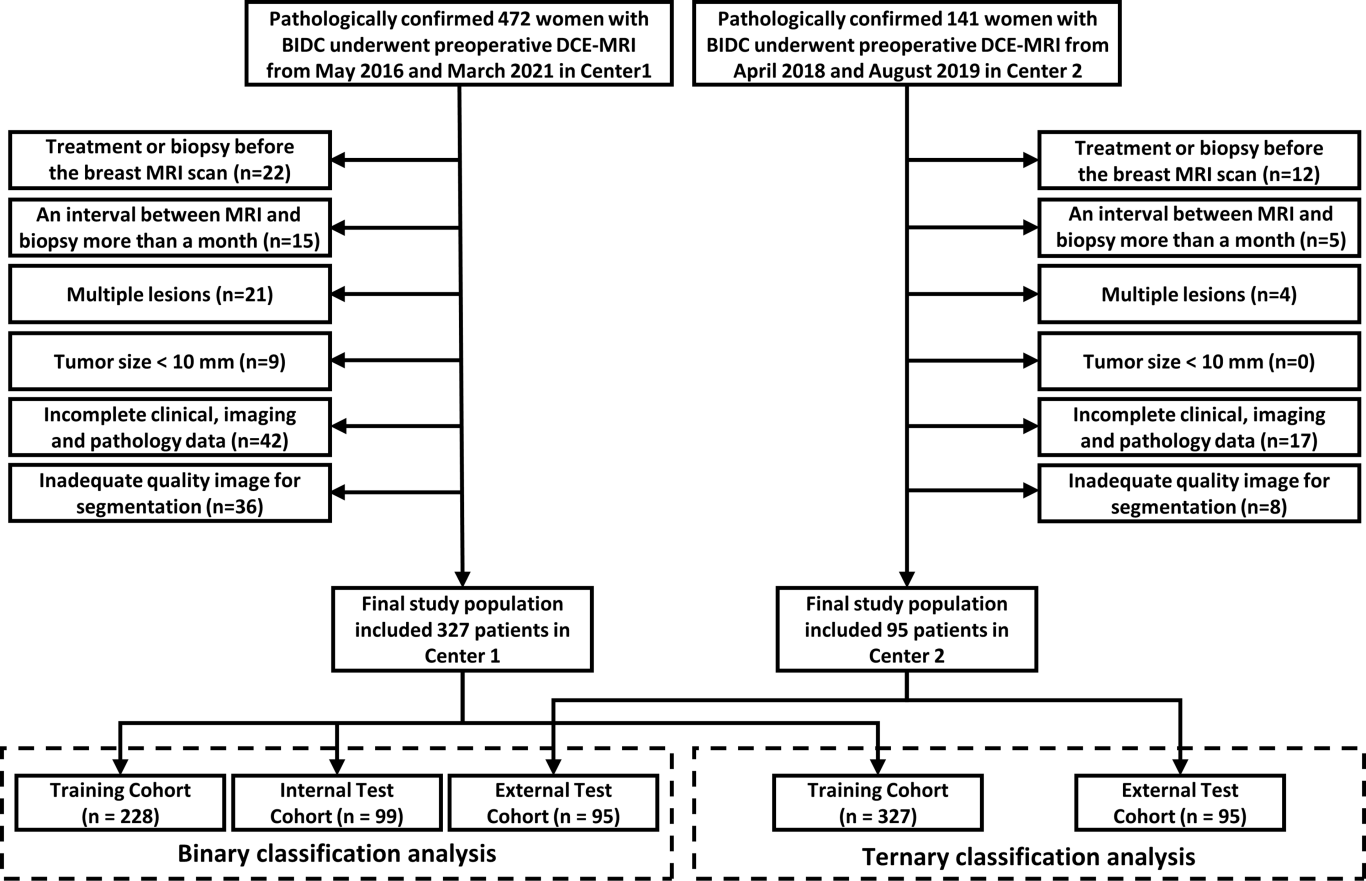
Flow diagram of study enrollment.

We finally included 422 women who met the criteria in this study (**Figure 1**). In the binary classification analyses, 327 women from Center 1 were randomly divided into two datasets at a ratio of 7:3 (228 in the training cohort and 99 in the internal test cohort). A total of 95 women from Center 2 were allocated to the external test cohort. To avoid overfitting in the ternary classification analysis, given the difference in sample size, all patients from Center 1 were allocated to the training cohort, and patients from Center 2 comprised the test cohort.

### Analysis of Molecular Subtypes

The expressions of ER, PR, and HER2 for each patient were recorded from IHC and fluorescence *in situ* hybridization (FISH) results. The molecular subtypes were diagnosed in accordance with the American Society of Clinical Oncology (ASCO)/College of American Pathologists (CAP) guidelines (27). When at least 1% of tumor cell nuclei exhibited positive staining for ER or PR, the samples were considered as ER-positive or PR-positive, respectively. For the expression status of HER2, a score of 3+ was defined as positive and a score of 0 or 1+ was considered negative. And in the case of HER2 scores between 1+ and 3+, samples will be further tested by FISH for a definite diagnosis. According to the expression status of ER, PR, and HER2, IDBC patients were subdivided into three molecular subtypes as HR-positive (ER+ and/or PR+, HER2+, or HER2-), HER2-enriched (ER-, PR-, and HER2+), and TNBC (ER-, PR-, and HER2-) (**Supplementary Table S1**). Three binary classification tasks (task 1 as HR-positive vs HR-negative; task 2 as HER2-enriched vs non-HER2-enriched; task 3 as TNBC vs non-TNBC) were performed for predicting the molecular subtypes. In addition, to further analyze the overall molecular subtype differences, a ternary classification task (task 4 as HR-positive vs HER2-enriched vs TNBC) was developed.

### MRI Examinations

For all patients enrolled in this study, images were obtained respectively with two MRI scanners, a 3.0-T MRI scanner (Philips Achieva, Center 1) and a 1.5-T scanner (GE Signa HD, Center 2). All patients were positioned in the prone position and scanned with a bilateral dedicated breast coil. T2WI, T1WI, diffusion-weighted imaging (DWI), pre-contrast-enhancement T1WI, and DCE were sequentially acquired. Gd-DTPA (Magnevist) was used as the contrast agent in both centers. Pre-contrast-enhancement T1WI was obtained prior to contrast agent injection. For the DCE sequence, six phases (Center 1) and seven phases (Center 2) were acquired after the end of high-pressure syringe injection with 60 s (Center 1) and 70 s (Center 2) per phase, respectively. In both centers, Gd-DTPA was intravenously injected at a dose of 0.1 mmol/kg and at a flow rate of 2 ml/s, and 20–30 ml of saline flush was subsequently injected at the same flow rate. Detailed parameters are listed in **Supplementary Table S2**.

### Radiomics Feature Processing

**Figure 2:**
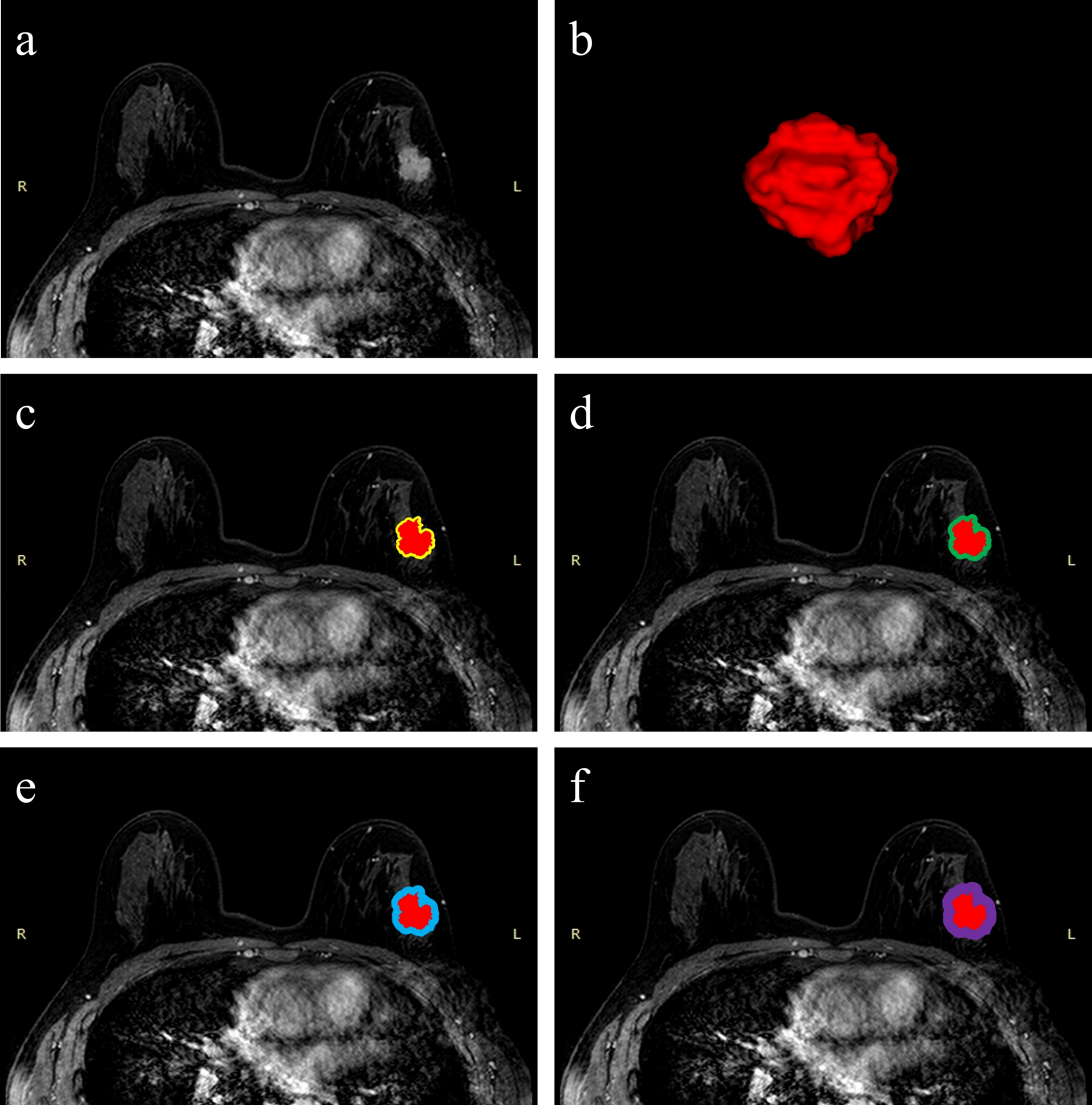
Schematic illustration of intratumoral and peritumoral ROI segmentation. The example segmentation is from Center 1, a 47-year-old woman diagnosed with a left-sided IDBC, 29 mm, Luminal B (HER2-positive). **(A)** Maximal cross-sectional of tumor on the first phase DCE-MRI image. **(B)** The 3D visualization intratumoral ROI with layer-by-layer delineation. **(C–F)** The red masks are the intratumoral ROIs, and the peritumoral ROIs are annular masks that automatically shape-modulated the intratumoral ROIs with sizes of 2 mm (yellow), 4 mm (green), 6 mm (blue), and 8 mm (purple).

As previous studies suggested, the contrast between the mass of breast cancer and background reached a peak at 60–120 s after contrast injection (28, 29). In this study, we selected the first phase of DCE-MRI from Center 1 and Center 2 to segment the region of interest (ROI). The normalization preprocessing of images is necessary before ROI segmentation since images are from two hospitals. Based on the slice thickness and pixel spacing, we performed image isotropic resampling and set new spacing to (1, 1, 1) to prevent image distortion caused by different scanners. The default window width and window level were read for each slice to get the average gray value of the whole 3D data, and the gray values of all slices were normalized to the range (1, 4097) using a linear transformation. After image preprocessing, intratumoral ROIs were annotated on a “DARWIN intelligent research platform” by two radiologists (Reader 1 SZ and Reader 2 XW) with more than five years of experience, and they were blinded to the patients’ clinical, radiological, and pathological data. The intratumoral ROIs were manually delimitated by Reader 1 slice by slice on the first phase of axial DCE-MRI images. Considering that the lesion boundaries are sensitive to partial-volume effects on MRI, the boundaries of the intratumoral ROI is slightly smaller than the lesion boundary observed by human eyes (30). After 1 month, 10% of the images were randomly selected from Center 1 and Center 2, and the intratumoral ROIs of the selected images were segmented again by Reader 1 and Reader 2 to calculate the inter-/intraclass correlation coefficient (ICC) for evaluating segmentation reproducibility. Since different peritumoral regions within 10 mm of the tumor may be closely associated with tumor vascular density, lymph node metastasis, and biomarkers, we set four peritumoral ranges of 2, 4, 6, and 8 mm, drawing on a previous peritumoral radiomics study (22, 31). The 2-, 4-, 6-, and 8-mm peritumoral ROIs are ring-like regions obtained by automatically expanding the intratumoral ROIs slice by slice (**Figure 2**). After segmentation, the three-dimensional ROIs of the intratumoral and peritumoral regions were obtained. To further amplify the abundance of features, the intratumoral and peritumoral ROIs in the original image underwent six image-filtering processes, including squared filtering, square root filtering, exponential filtering, logarithm filtering, Laplacian of Gaussian (LOG) filtering, and wavelet filtering. Finally, a total of 1,316 radiomics features were automatically extracted over the original and derived filtered ROIs based on an open-source Python package: Pyradiomics (https://pyradiomics.readthedocs.io).

To prevent the influence of the magnitude difference between features on feature selection, we normalized the feature magnitude before feature dimensionality reduction. All features were transformed to between (-1, 1) by using the maximum absolute value normalization method. In the first feature selection step, features were retained if both inter-/intraclass correlation coefficients > 0.75. Then, a statistical feature selection method named “Select K Best” with f_calssif function were used to further reduce feature dimensionality. The threshold for K was set to 20 while calculating the F statistics of ANOVA and retaining the features with F values in the top 20%. Finally, an embedded feature selection method, L1-regularized support vector machine (SVM-L1), was used to filter out the optimal radiomics features. More details about image filtering and feature normalization processing are given in the Supplementary Materials and Methods.

### Clinical–Radiological Characteristics

Clinical characteristics including age, mass palpation (firmness and mobility), and menopausal status were obtained by reviewing the patient’s clinical records. Radiological characteristics including tumor size, background parenchymal enhancement (BPE), fibro glandular tissue (FGT), margin sharpness, and short diameter of axillary lymph node (ALN) were evaluated mainly by two other radiologists (Reader 3 ZY with 4 years of experience in MRI diagnosis, and Reader 4 YZ with 6 years of experience). In cases of disagreement, Reader 5 (ZX) with 14 years of experience made the final decision. After univariate and multivariate logistic regression analyses, the selected characteristics were used to be the clinical–radiological independent predictors for predicting molecular subtypes in the four classification tasks. More details about the clinical–radiological characteristics processing are given in the Supplementary Materials and Methods.

### Model Construction and Validation

**Figure 3:**
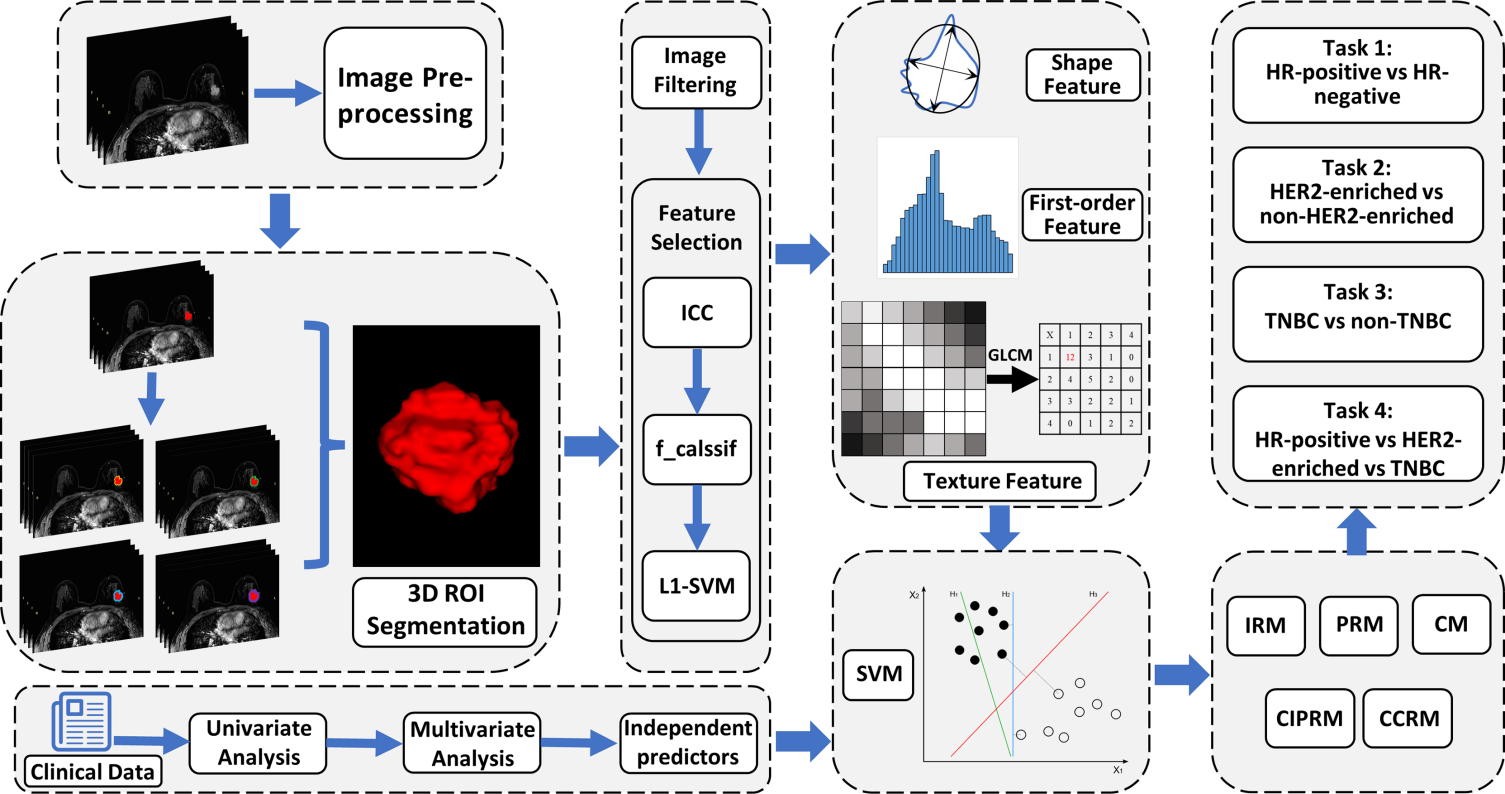
An overview of radiomics analysis methodology in this study. Five predictive models were developed based on clinical–radiological characteristics and intratumoral and peritumoral radiomics features. CM, clinical–radiological model; IRM, intratumoral radiomics model; PRM, peritumoral radiomics model; CIPRM, combined intratumoral and peritumoral radiomics model; CCRM, combined clinical–radiological and radiomics model.

An overview of the radiomics analysis pipeline is shown in **Figure 3**. SVM was used as a classifier to construct the intratumoral radiomics model (IRM) and 2-, 4-, 6-, and 8-mm peritumoral radiomics model (PRM) with the selected radiomics features, and Rad-scores for each model were calculated. In tasks 1–3, the receiver operating characteristic (ROC) curves were plotted to evaluate the performance of each PRM, and the area under the ROC curves (AUC) was performed to select the optimal PRM. In task 4, a confusion matrix was plotted to calculate the accuracy of PRM, and the optimal PRM was selected. Subsequently, the combined intra- and peritumoral radiomics model (CIPRM) was developed based on the intratumoral and optimal peritumoral Rad-score. Clinical–radiological independent predictors in the training cohort were used to establish clinical–radiological models (CMs) by logistic regression. Finally, we used the intratumoral Rad-score, the optimal peritumoral Rad-score, and clinical–radiological independent predictors to develop the combined clinical–radiological and radiomics models (CCRMs).

In tasks 1–3, the ROCs were plotted to assess the performance of each model, and DeLong test was used to select the optimal model and to evaluate the consistency of the model in the training cohort, the internal test cohort, and the external test cohort. Tenfold cross-validation was applied to evaluate model stability. Nomogram, calibration curve, and decision curve analysis (DCA) were depicted to visualize models, evaluate model fit, and analyze clinical usefulness, respectively. In task 4, a confusion matrix was used to describe the performance of ternary classification models.

### Statistical Analysis

All statistical analyses were performed using the SPSS 22.0 software (SPSS, Chicago, IL) and R software (Version 4.1.0). Kolmogorov–Smirnov test was used to test the data normality. In binary classification tasks, independent-sample t-test, Mann–Whitney U test, and binary logistic regression analysis were used to assess the association between the molecular subtypes and features. And in the ternary classification task, ANOVA, Kruskal–Wallis test, and multinomial logistic regression analysis were used to analyze the association between the molecular subtypes and features. ICC > 0.75 was considered to indicate good reproducibility of segmentation. Delong test was used to compare the differences of ROCs. P < 0.05 was considered statistically significant.

## Results

### Patient Characteristics

A total of 422 patients from Center 1 (327 cases, 49.61 ± 9.36 years) and Center 2 (95 cases, 51.55 ± 9.15 years) were included in this study. HR-positive is the most common subtype and comprised 69.4% of cases (293/422), and HER2-enriched was the least common accounting for 14.0% of cases (59/422). The difference of clinical–radiological and pathological characteristics in the training, internal, and external test cohorts is not statistically significant different. The detailed clinical–radiological and pathological characteristics are described in **Table 1**. And the clinical–radiological independent predictors in tasks 1–4 are shown in **Supplementary Figure S1** and **Supplementary Table S3**. The logistic regression analysis showed that a short diameter of the axillary lymph node (ALN) (P < 0.001, OR = 0.264, 95% confidence interval [CI]: 0.146–0.479) and mass palpation (mobility) (P = 0.018, OR = 0.376, 95% CI: 0.168–0.843) were selected as independent predictors in task 1; margin sharpness (P = 0.025, OR = 3.146, 95% CI: 1.158–8.547) and short diameter of ALN (P = 0.004, OR = 2.374, 95% CI:1.312–4.298) were selected as independent predictors in task 2; the short diameter of ALN (P = 0.003, OR = 2.886, 95% CI:1.436–5.800) was selected as an independent predictor in task 3. For task 4, tumor size was selected as an independent predictor.

**Table 1:** Clinical–radiological and pathological characteristics of patients in the training, internal, and external cohorts.

Characteristics		All patients	Training	Internal test	External test	statistics	P
Number		422	228	99	95	–	–
Molecular subtypes^c^						8.791^d^	0.186
	HR-positive	293 (69.4%)	153 (67.1%)	67 (67.7%)	73 (76.8%)		
	HER2-enriched	59 (14.0%)	32 (14.0%)	18 (18.2%)	9 (9.5%)		
	TNBC	70 (16.6%)	43 (18.9%)	14 (14.1%)	13 (13.7%)		
Age^a^		50.05 ± 9.33	49.71 ± 8.89	49.39 ± 10.39	51.54 ± 9.15	2.218^e^	0.11
Tumor size^b^		21 (16-28)	21 (16-28)	22 (16-30)	20 (15-25)	5.899^d^	0.052
BPE^c^						0.434^d^	0.805
	Minimal	18 (4.3%)	10 (4.4%)	3 (3%)	5 (5.3%)		
	Mild	228 (54%)	131 (57.5%)	48 (48.5%)	49 (51.6%)		
	Moderate	123 (29.1%)	63 (27.6%)	30 (30.3%)	30 (31.6%)		
	Marked	53 (12.6%)	24 (10.5%)	18 (18.2%)	11 (11.6%)		
FGT^c^						0.095^d^	0.954
	Dense	120 (28.4%)	66 (28.9%)	27 (27.3%)/	27 (28.4%)		
	Non-dense	302 (71.6%)	162 (71.1%)	72 (72.7%)	68 (71.6%)		
Margin sharpness^c^						2.567^d^	0.168
	Clear	84 (19.9%)	45 (19.7%)	14 (14.1%)	25 (26.3%)		
	Blurry	338 (80.1%)	183 (80.3%)	85 (85.9%)	70 (73.7%)		
Short diameter of ALN^c^						5.007^d^	0.082
	≤ 5 mm	282 (66.8%)	149 (65.4%)	61 (61.6%)	71 (74.7%)		
	> 5 mm	140 (33.2%)	79 (34.6%)	38 (38.4%)	24 (25.3%)		
Mass palpation (Firmness)^c^						5.886^d^	0.053
	Soft	67 (15.9%)	33 (14.5%)	17 (17.2%)/	17 (17.9%)		
	Hard	355 (84.1%)	195 (85.5%)	82 (82.8%)	78 (82.1%)		
Mass palpation (Mobility) ^c^						1.091^d^	0.58
	Pushable	112 (26.5%)	53 (23.2%)	30 (30.3%)	29 (30.5%)		
	Non-pushable	310 (73.5%)	175 (76.8%)	69 (69.7%)	66 (69.5%)		
Menopausal Status^c^						2.482^d^	0.289
	Premenopausal	216 (51.2%)	117 (51.3%)	57 (57.6%)	42 (44.2%)		
	Postmenopausal	206 (48.8%)	111 (48.7%)	42 (42.4%)	53 (55.8%)		

Characteristics^a^: The measurement data conforming to the normal distribution were expressed as mean ± standard deviation.

Characteristics^b^: The non-normally distributed measurement data were expressed as median (lower quartile-upper quartile).

Characteristics^c^: The enumeration data were expressed as frequency (constituent ratio); Statistic ^d^: H-value; Statistic ^e^: F-value; P is derived from univariate association analyses between each of the Patients Characteristics and Molecular subtypes, and P < 0.05 is considered statistically significant. ALN, axillary lymph node; BPE, background parenchymal enhancement; FGT fibro glandular tissue; HER2, human epidermal growth factor receptor 2; HR hormone receptor; TNBC triple-negative breast cancer.

### Radiomics Features

Out of the 1,316 extracted radiomics features from the intratumor, 82.8% (1,089 features) were retained when ICCs > 0.75. The peritumor retained the same 1,089 features as the intratumor. Based on the predicted labels for tasks 1–4, 217 features were retained by the f_calssif function. After being filtered by SVM-L1, optimal intra-/peritumoral radiomics features were selected to construct the classification models (**Table S4**). Among the selected features, features based on the wavelet filtering images occupied the highest proportion (49.30%, 35:71), followed by features based on original images (21.13%, 15:71).

### Optimal Peritumoral Model Selection

**Table 2** summarizes the performance of PRMs in binary classification tasks (tasks 1–3). The 6-mm PRM was selected as the optimal peritumoral size in task 1 (AUC: 0.794, 95% CI: 0.735–0.844) and task 3 (AUC: 0.906, 95% CI: 0.860–0.941). And the 8-mm PRM achieved the highest AUC (0.784, 95% CI: 0.741–0.823) in task 2. In task 4, the 6-mm peritumoral model showed the highest accuracy (0.697) from the confusion matrix.

**Table 2:** Performance of peritumoral models in tasks 1–3.

Task	Peritumoral Size	AUC (95%CI)	Sensitivity	Specificity
Task 1	2 mm	0.681 (0.616-0.741)	73.86	61.33
	4 mm	0.725 (0.662-0.782)	71.90	69.33
	6 mm	0.794 (0.735-0.844)	63.41	86.67
	8 mm	0.751 (0.689-0.805)	77.12	65.33
Task 2	2 mm	0.642 (0.576-0.704)	55.56	70.51
	4 mm	0.744 (0.683-0.800)	66.67	72.44
	6 mm	0.728 (0.665-0.785)	69.44	69.87
	8 mm	0.784 (0.741-0.823)	79.84	65.23
Task 3	2 mm	0.787 (0.728-0.838)	95.01	54.26
	4 mm	0.751 (0.690-0.806)	80.00	60.64
	6 mm	0.906 (0.860-0.941)	98.62	69.5
	8 mm	0.769 (0.709-0.822)	72.5	76.06

Task 1: Classification task for prediction of HR-positive and HR-negative. Task 2: classification task for prediction of HER2-enriched and non-HER2-enriched. Task 3: classification task for the prediction of TNBC and non-TNBC. AUC, Area under the curve; CI, Confidence interval.

### Model Performance

**Figure 4:**
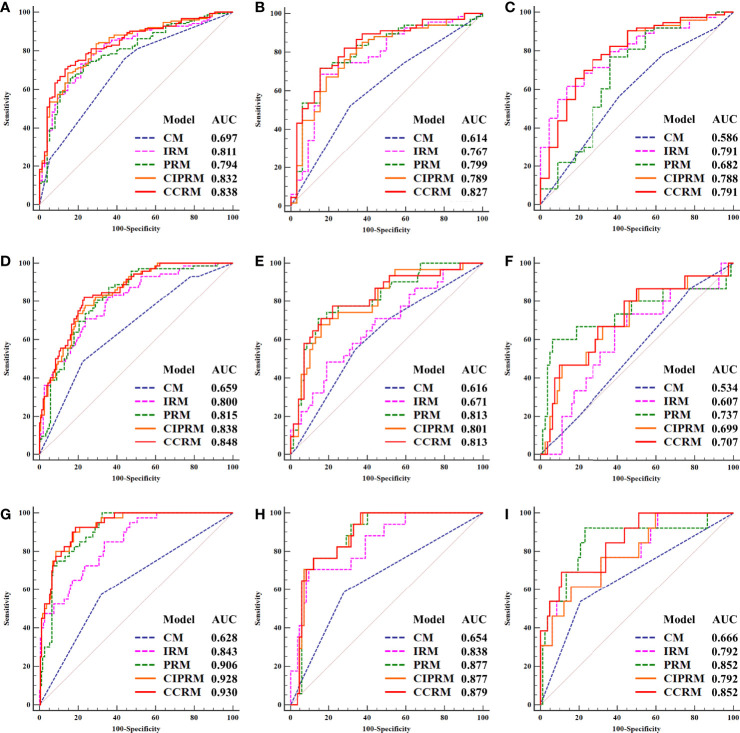
Receiver operating characteristic (ROC) curves of five models in the training, internal, and external test cohorts of task 1 (HR-positive vs HR-negative, **(A)** training cohort, **(B)** internal test cohort, **(C)** external test cohort), task 2 (HER2-enriched vs non-HER2-enriched, **(D)** training cohort, **(E)** internal test cohort, **(F)** external test cohort), and task 3 (TNBC vs non-TNBC, **(G)** training cohort, **(H)** internal test cohort, **(I)** external test cohort).

**Figure 5:**
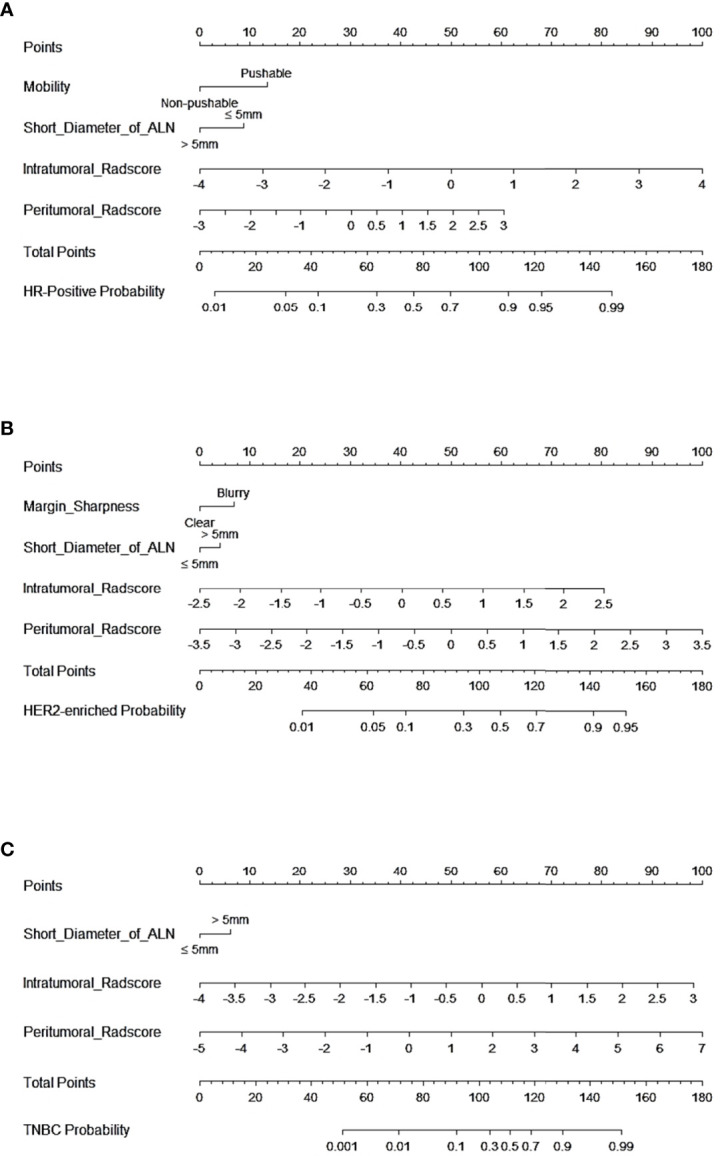
Nomogram of CCRMs in tasks 1–3. **(A)** Nomogram of CCRMs for the prediction of HR-positive and HR-negative in task 1. **(B)** Nomogram of CCRMs for the prediction of HER2-enriched and non-HER2-enriched in task 2. **(C)** Nomogram of CCRMs for the prediction of TNBC and non-TNBC in task 3.

**Figure 6:**
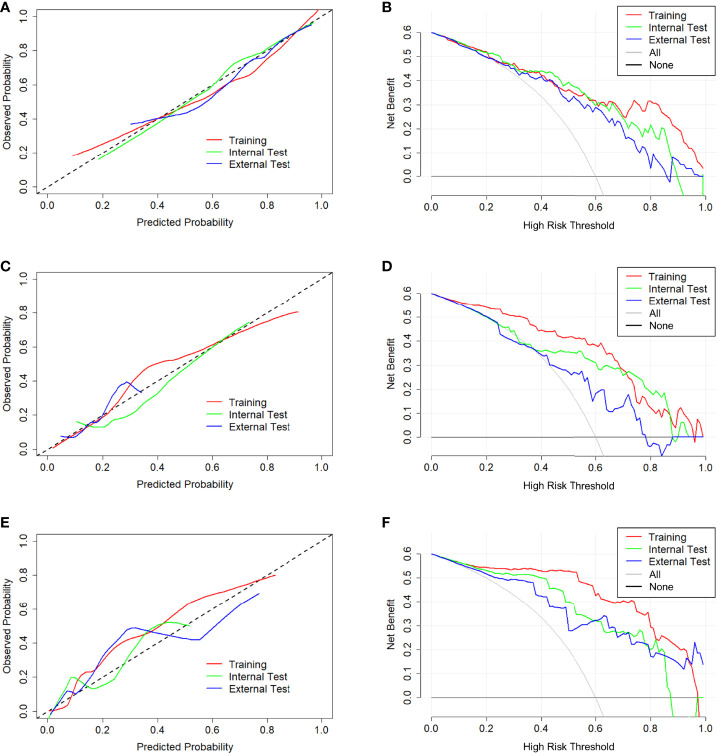
Calibration curve and decision curve of CCRMs in tasks 1–3. Calibration curve **(A)** and decision curve **(B)** of CCRMs for the prediction of HR-positive and HR-negative in task 1. Calibration curve **(C)** and decision curve **(D)** of CCRMs for the prediction of HER2-enriched and non-HER2-enriched in task 2. Calibration curve **(E)** and decision curve **(F)** of CCRMs for the prediction of TNBC and non-TNBC in task 3. In the calibration curve, the gray dotted line represents the ideal line of the model. In decision curves, the gray solid line represents the net benefit for all patients after pathological diagnosis (All), and the black solid line represents the net benefit for none patients after pathological diagnosis (None).

**Figure 7:**
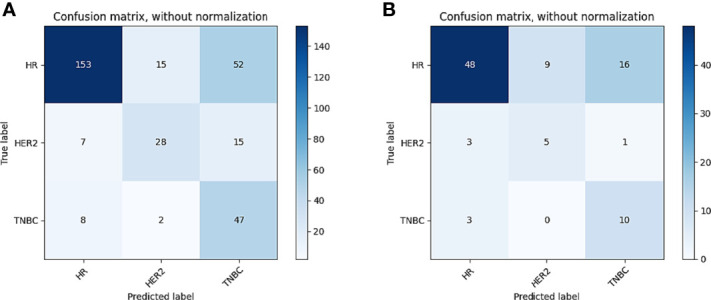
Confusion matrix of CIPRM in task 4. **(A)** Confusion matrix of the training cohort; **(B)** confusion matrix of the test cohort.

In tasks 1–3, DeLong test showed that the models combined with peritumoral radiomics features exhibited higher performance compared to the individual models (**Table 3**). The CCRMs yielded optimal performance, and ROC analyses showed that the AUCs were 0.838, 0.827, and 0.791 in training, internal test, and external test cohorts of task 1, respectively; 0.848, 0.813, and 0.707 in task 2, respectively; 0.930, 0.879, and 0.852 in task 3, respectively (**Table 4** and **Figure 4**). Internal and external tests proved that the models had satisfactory repeatability. After the 10-fold cross-validation, CCRMs performed excellent stability (**Supplementary Figure S2**). In addition, DeLong test showed that the CCRMs have good consistency in the training, internal, and external test cohorts of tasks 1–3 (P > 0.05, **Table 5**). The nomograms of the CCRMs showed that intratumoral and peritumoral Rad-scores were given higher weighting compared to the clinical–radiological independent predictors (**Figure 5**). The calibration curves illustrated that CCRMs are in excellent agreement with the ideal curve, and the DCA demonstrated that CCRMs have a high overall net benefit (**Figure 6**). In task 4, CIPRM instead of CCRM had the highest accuracy (training cohort: 0.697; test cohort: 0.663) and the highest F1-score (training cohort: 0.79; test cohort: 0.76) from the confusion matrix among the five models (**Figure 7** and **Supplementary Table S5**, **Supplementary Figure S3**).

**Table 3:** Pairwise comparison of ROC curves in tasks 1–3.

Models	Task 1		Task 2		Task 3	
	Z Statistic	P	Z Statistic	P	Z Statistic	P
CM vs IRM	2.821	0.005*	3.394	< 0.001*	3.908	< 0.001*
CM vs PRM	2.271	0.023*	3.855	< 0.001*	5.947	< 0.001*
CM vs CIPRM	3.408	< 0.001*	4.799	< 0.0001*	6.315	< 0.001*
CM vs CCRM	4.093	< 0.001*	5.296	< 0.0001*	6.476	< 0.001*
IRM vs PRM	0.591	0.554	0.409	0.683	1.879	0.06
IRM vs CIPRM	1.551	0.121	1.512	0.131	3.267	0.001*
IRM vs CCRM	1.775	0.076*	1.964	0.05*	3.388	< 0.001*
PRM vs CIPRM	2.197	0.028*	1.755	0.079*	1.992	0.046*
PRM vs CCRM	2.467	0.014*	2.516	0.012*	2.073	0.038*
CIPRM vs CCRM	0.9	0.368	1.495	0.135	0.74	0.459

P is derived from Delong test between each of the models, and P* < 0.05 is considered statistically significant. CM, clinical–radiological model; CIPRM, combined intra- and peritumoral radiomics model; CCRM, combined clinical–radiological and radiomics model; IRM, intratumoral radiomics model; PRM, peritumoral radiomics model.

**Table 4:** Summary of five models’ performance in training, internal test, and external test cohorts of tasks 1–3.

Task	Models	Training Cohort			Internal Test Cohort			External Test Cohort		
		AUC (95%CI)	Sensitivity	Specificity	AUC (95%CI)	Sensitivity	Specificity	AUC (95%CI)	Sensitivity	Specificity
Task1	CM	0.697 (0.633-0.756)	75.82%	56%	0.614 (0.511-0.710)	52.24%	68.75%	0.586 (0.481-0.686)	56.16%	59.09%
	IRM	0.811 (0.754-0.859)	73.20%	78.67%	0.767 (0.672-0.846)	68.66%	84.37%	0.791 (0.696-0.868)	61.64%	86.36%
	PRM	0.794 (0.735-0.844)	63.40%	86.67%	0.799 (0.706-0.873)	71.64%	84.37%	0.682 (0.578-0.774)	76.71%	63.64%
	CIPRM	0.832 (0.777-0.878)	83.66%	70.67%	0.789 (0.695-0.864)	67.16%	81.25%	0.788 (0.692-0.865)	75.34%	72.73%
	CCRM	0.838 (0.784-0.884)	70.59%	86.67%	0.827 (0.738-0.896)	71.64%	84.37%	0.791 (0.696-0.868)	75.34%	72.73%
Task2	CM	0.659 (0.593-0.720)	48.61%	77.56%	0.616 (0.513-0.712)	54.84%	66.18%	0.534 (0.429-0.637)	22.50%	86.67%
	IRM	0.800 (0.742-0.850)	70.83%	76.28%	0.671 (0.569-0.762)	48.39%	80.88%	0.607 (0.501-0.705)	55%	73.33%
	PRM	0.815 (0.758-0.863)	87.50%	64.10%	0.813 (0.722-0.884)	70.97%	85.29%	0.737 (0.636-0.822)	93.75%	60.00%
	CIPRM	0.838 (0.784-0.884)	77.78%	76.28%	0.801 (0.708-0.874)	67.74%	83.82%	0.699 (0.596-0.789)	48.75%	86.67%
	CCRM	0.848 (0.795-0.892)	81.94%	76.92%	0.813 (0.722-0.884)	77.42%	77.94%	0.707 (0.604-0.796)	50%	86.67%
Task3	CM	0.628 (0.562-0.691)	57.50%	68.09%	0.654 (0.552-0.747)	58.82%	71.95%	0.666 (0.561-0.759)	53.85%	79.27%
	IRM	0.843 (0.790-0.888)	85%	66.49%	0.838 (0.750-0.904)	70.59%	90.24%	0.792 (0.696-0.868)	61.54%	84.15%
	PRM	0.906 (0.860-0.941)	100%	67.55%	0.877 (0.795-0.934)	76.47%	87.80%	0.852 (0.764-0.916)	92.31%	76.83%
	CIPRM	0.928 (0.887-0.958)	90%	82.45%	0.877 (0.795-0.934)	76.47%	87.80%	0.792 (0.696-0.868)	61.54%	84.15%
	CCRM	0.930 (0.888-0.959)	92.50%	81.38%	0.879 (0.799-0.936)	76.47%	87.80%	0.852 (0.764-0.916)	69.23%	89.02%

AUC, area under the curve; CI, confidence interval; CM, clinical–radiological model; CIPRM, combined intra- and peritumoral radiomics model; CCRM, combined clinical–radiological and radiomics model; IRM, intratumoral radiomics model; PRM, peritumoral radiomics model.

**Table 5:** DeLong Test between Training Cohort, Internal Test Cohort, and External Test Cohort in tasks 1–3.

Tasks	Compares	Z	P
Task 1	External Test Cohort vs Internal Test Cohort	0.514	P = 0.6075
	External Test Cohort vs Training Cohort	0.772	P = 0.4400
	Internal Test Cohort vs Training Cohort	0.216	P = 0.8292
Task 2	External Test Cohort vs Internal Test Cohort	1.154	P = 0.2485
	External Test Cohort vs Training Cohort	1.711	P = 0.0871
	Internal Test Cohort vs Training Cohort	0.634	P = 0.5260
Task 3	External Test Cohort vs Internal Test Cohort	0.41	P = 0.6821
	External Test Cohort vs Training Cohort	1.32	P = 0.1868
	Internal Test Cohort vs Training Cohort	1.193	P = 0.2329

P is derived from Delong test between each of the ROCs, and P < 0.05 is considered statistically significant.

## Discussion

In this multitask radiomics analysis, we developed the noninvasive radiomics models based on DCE-MRI to preoperatively predict molecular subtypes of IDBC. The subtype classification results of both the individual models and combined models showed reasonable distinguishing performance. And the CCRMs achieved excellent predictive performance for predicting molecular subtypes in the binary classification tasks. In contrast to an individual clinical–radiological model or a radiomics model, the combined model with optimized peritumoral radiomics features has higher predictive values for the molecular subtypes of IDBC.

The microenvironment surrounding breast cancer has been reported to contain important biological information that may result in subtle changes on MRI images (20, 22, 32, 33). However, the optimal peritumoral area size for breast cancer on DCE-MRI remains controversial. Wang et al. found that the radiomics features extracted from the 3-mm peritumoral region could differentiate benign and malignant breast lesions on contrast-enhanced mammography (25). Li et al. extracted intratumoral and 4-mm peritumoral radiomics features to predict the expression of HER2 and Ki-67 in breast cancer, and their models achieved good performance (26). In our study, 6 mm was determined as the optimal peritumoral size in tasks 1, 3, and 4, and 8 mm was determined as the optimal peritumoral size in task 2. In agreement with our findings, Ding et al. also analyzed radiomics features of multiple peritumoral areas, and they found that 4-, 6-, and 8-mm peritumoral radiomics features could further improve the performance of lymph node metastasis radiomics models in breast cancer (31). Our results demonstrate the excellent prediction performance of peritumoral radiomics features and suggest that the optimal peritumoral size should be selected based on the predictive label, which could further optimize the performance of the model.

The SVM is one of most frequently used machine-learning methods for solving classification problems, which is not susceptible to feature colinearity and is not prone to overfitting (34). In this study, we used the SVM to construct several high-performance and stable radiomics models. In tasks 1–3, CCRMs exhibited higher performance compared to other models. After internal test, external test, and 10-fold cross-validation, CCRMs showed satisfactory repeatability and stability. In the three binary classification tasks, the CCRM identifying TNBC vs non-TNBC exhibited the highest AUC. Our results are consistent with previous studies. Son et al. constructed three models for predicting molecular subtypes of breast cancer based on digital breast tomosynthesis (DBT), and they suggested that the radiomics features were superior for predicting TNBC over HER2 and luminal-like subtypes (AUC: 0.838, 0.556, and 0.645) (35). Doris et al. reported that radiomics features extracted from multiparametric MRI could noninvasively assess breast cancer molecular subtypes (Accuracy: 0.852, AUC: 0.860) (15). Compared to these results, the binary classification models developed in our study exhibit better differentiable capability, and this may be due to the fact that our study is based on 3D segmentation and optimized the peritumoral ROIs, which could obtain more comprehensive tumor information.

Comparatively, the model performance was not adequate in task 4 (training cohort: 0.697; test cohort: 0.663). The result is in agreement with a recent study by Huang et al. who built ternary classification MRI-based models to predict molecular subtypes of breast cancer (accuracy, 0.623–0.735) (36). Although we incorporated the optimal peritumoral features, the performance is still unsatisfactory in the ternary classification task. We argue that the molecular heterogeneity of the IDBC may lead to the diversification of radiomics features, which could be amplified in the ternary classification model resulting in reduced performance. Particularly, the optimal model (CIPRM) in task 4 did not incorporate a clinical–radiological feature, which was the result of the collinearity that exists between the radiomics shape feature maximum 2D diameter and the clinical–radiological feature tumor size.

Previous studies have reported that voxel size resampling and gray level normalization could reduce the variability of radiomics features (37, 38). In our study, we validated the radiomics-based classification models for evaluating the generalization ability of the models by external test cohort from center 2. As different centers have different protocols for imaging, we utilized image resampling and normalization before ROI segmentation to help mitigate the effects of data heterogeneity. After pairwise comparisons of the training cohort, the internal test cohort, and the external test cohort, Delong test showed that the ROC of CCRM was not statistically significantly different between the three groups. This result may indicate that the difference of imaging protocols in the two centers had a negligible effect on the models constructed after image preprocessing. However, the external test cohort revealed relatively low AUC compared to other groups, which may be due to the fact that image preprocessing did not completely eliminate the differences of imaging protocols in the two centers, and the sample size of the external test cohort was relatively small.

Moreover, our results suggest that there might be unique radiomics features in intratumoral and peritumoral regions associated with microscopic morphological alterations in molecular subtypes. Among the radiomics features included in this study, wavelet features accounted for the highest proportion, which may suggest that the features from wavelet-filtered DCE-MRI potentially associated with IDBC molecular subtypes. In consistent with our results, Li et al. extracted wavelet features for predicting HER2 and Ki-67 expression status and suggested that wavelet features contain more detailed information about breast cancer (26). In addition, second- and higher-order texture features accounted for a higher proportion (56.34%) compared to shape features and first-order statistical features, suggesting that regional or local variation predominates over global variation in the distribution of different molecular subtypes (39).

Several limitations exist in this study. First, this is a retrospective study, leading to inevitable selection bias. Moreover, molecular subtypes are derived from IHC results at two hospitals but not from formal genetic testing, which might result in unstable results from the external test cohort. Third, in our study, the proportion of the peritumoral size was not adjusted for the size of the tumor itself. More detailed subgroup analysis according to different tumor sizes should be further studied to illustrate the wide applicability of the tumor outer margin. Finally, this study only constructed the radiomics models based on the first phase of DCE-MRI and did not analyze the whole DCE-MRI sequence, and further radiomics research on different phases of DCE-MRI is needed in the future.

In conclusion, this study further demonstrates the feasibility of preoperative radiomics analysis in predicting the molecular subtypes of IDBC. Combining the radiomics features of the intratumoral and the optimal peritumoral region from early DCE-MRI could effectively predict the HR-positive, HER2-enriched, and TNBC molecular subtypes of IDBC and potentially provide guidance for preoperative clinical decision-making.

## Data Availability Statement

The original contributions presented in the study are included in the article/**Supplementary Material**. Further inquiries can be directed to the corresponding authors.

## Ethics Statement

Written informed consent was not obtained from the individual(s) for the publication of any potentially identifiable images or data included in this article.

## Author Contributions

Guarantor of integrity of the entire study: ZX and HS. Study concepts and design: SZ. Literature research: XW, ZY, and NZ. Data acquisition: SZ, JH, NZ, and YL. Clinical studies: YZ, XW, and ZX. Data analysis and interpretation: SZ and XW. Statistical analysis: SZ and XW. Manuscript preparation: SZ. Manuscript editing: SZ and HS. All authors contributed to the article and approved the submitted version.

## Funding

This study has received funding by Key Project of Natural Science Research in Anhui Universities (KJ2019A0402), “Science and Technology Innovation Action Plan” Star Cultivation (Sailing Program) (22YF1443600), and Shanghai Municipal Key Clinical Specialty (shslczdzk03202).

## Conflict of Interest

The authors declare that the research was conducted in the absence of any commercial or financial relationships that could be construed as a potential conflict of interest.

## Publisher’s Note

All claims expressed in this article are solely those of the authors and do not necessarily represent those of their affiliated organizations, or those of the publisher, the editors and the reviewers. Any product that may be evaluated in this article, or claim that may be made by its manufacturer, is not guaranteed or endorsed by the publisher.
