# Supplementary material for: Intra- and Peritumoral Radiomics Model Based on Early DCE-MRI for Preoperative Prediction of Molecular Subtypes in Invasive Ductal Breast Carcinoma: A Multitask Machine Learning Study
Source: Front Oncol. 2022 Jun 24;12:905551. doi: 10.3389/fonc.2022.905551 (PMC9263840; doi:10.3389/fonc.2022.905551)
Supplement: Supplementary file 1 [file DataSheet_1.docx]

Supplementary Material

# Supplementary Data

## Image Filtering Processing

To further amplify the abundance of features, we filtered the original image with six image filtering processes ^[1]^. The image filtering processes are indicated below.

1. Square filter

Computes the square of the image intensities. Resulting values are rescaled on the range of the initial original image and negative intensities are made negative in resultant filtered image.

$$f\left( x \right)=\left( cx \right)^{2}, where c=\frac{1}{\sqrt{\max\left( \left| x \right| \right)}}$$

Where $x$ and $f\left( x \right)$ are the original and filtered intensity, respectively.

1. Square Root filter

Computes the square root of the absolute value of image intensities. Resulting values are rescaled on the range of the initial original image and negative intensities are made negative in resultant filtered image.

$$f\left( x \right)=\left\{ \begin{aligned} \sqrt{cx} x>0 \\ -\sqrt{-cx} x<0 \end{aligned} \right. c=\max\left( \left| x \right| \right)$$

Where $x$ and $f\left( x \right)$ are the original and filtered intensity, respectively.

1. Exponential filter

Computes the exponential of the original image. Resulting values are rescaled on the range of the initial original image.

$$f\left( x \right)=e^{cx}, where c=\frac{\log\left( max\left| x \right| \right)}{\max\left( \left| x \right| \right)}$$

Where $x$ and $f\left( x \right)$ are the original and filtered intensity, respectively.

1. Logarithm filter

Computes the logarithm of the absolute value of the original image + 1. Resulting values are rescaled on the range of the initial original image and negative intensities are made negative in resultant filtered image.

$$f(x)\left\{ \begin{aligned} c\cdot log\left( x+1 \right) x>0 \\ -c\cdot\log\left( -x+1 \right) x<0 \end{aligned}, where c=\frac{\max\left( \left| x \right| \right)}{log(\max(\left| x \right|)+1)} \right.$$

Where $x$ and $f\left( x \right)$ are the original and filtered intensity, respectively.

1. Laplacian of Gaussian filter (LOG)

Applies a Laplacian of Gaussian filter to the input image and yields a derived image for each sigma value specified. The operator consists of two parts, a Gaussian kernel and a Laplace kernel:

$$G\left( x,y,z,\sigma\right)=\frac{1}{\left( \sigma\sqrt{2\pi} \right)^{3}}e^{-\frac{x^{2}+y^{2}+z^{2}}{2\sigma^{2}}}$$

The Gaussian kernel is convolved by the Laplacian kernel $\nabla2G(x, y, z)$, which is sensitive to areas with rapidly changing intensities, enhancing edges. The width of the filter in the Gaussian kernel is determined by $\sigma$ and can be used to emphasize more fine (low $\sigma$ values) or coarse (high $\sigma$ values) textures.

1. Wavelet filter

Applies wavelet filter to the input image and yields the decompositions and the approximation.

Wavelet Coiflets1 transform was used in this study, and its scaling function (low-pass filter) and wavelet function (high-pass filter) are orthogonal. In the three-dimensional images, the two filters were filtered separately for the x, y, z directions. And the following 8 forms were obtained from permutations and combinations:

Wavelet-LLL, Wavelet-LLH, Wavelet-LHL, Wavelet-LHH, Wavelet-HLL, Wavelet-HLH, Wavelet-HHL, Wavelet-HHH

## Feature Normalization Processing

To prevent differences in the magnitude of the radiomics feature values from affecting feature selection and model construction, we performed maximum absolute value normalization process to normalize the features. And the method is as follows:

$$f\left( x \right)=\frac{x}{\left| x \right|_{max}}$$

Where $x$ and $f\left( x \right)$ are the values of features before and after normalization, respectively. After normalization, all features were transformed to between (-1, 1).

## Evaluation of Clinical-Radiological Characteristics

Clinical characteristics including age, mass palpation (firmness and mobility) and menopausal status were obtained by reviewing the patient's clinical records. For mass palpation (firmness and mobility), we described tumor firmness as soft and hard, and the tumor mobility was described as pushable and non-pushable according to the palpation records.

Radiological characteristics including tumor size, background parenchymal enhancement (BPE), fibro glandular tissue (FGT), margin sharpness and short diameter of axillary lymph node (ALN) ^[2]^. Tumor size was defined as maximal diameter of tumor measured on the first phase of axial DCE-MRI images; BPE was obtained on the first phase of DCE-MRI images, the BPE is divided into four degrees according to the area of parenchymal enhancement: minimal, < 25%; mild, 25% ~ 50%; moderate, 50% ~ 75%; marked, >75%; FGT was analyzed on the pre-contrast-enhancement T1WI images and was divided into two types with reference to the percentage of the breast area occupied by fibro glandular tissue: dense, ≤ 50% and non-dense, >50%; Margin sharpness emphasis on the relationship of the lesion to the surrounding tissue. Based on the first phase of DCE-MRI images, margin sharpness is described as clear and blurry; Short diameter of ALN is measured on the sagittal and axial DCE-MRI images to divide two types: ≤ 5 mm and > 5 mm.

## References

1. van Griethuysen JJM, Fedorov A, Parmar C, et al. Computational Radiomics System to Decode the Radiographic Phenotype. Cancer Res 2017; 77:e104–e107.
2. Morris EA, Comstock CE, Lee CH, et al. ACR BI-RADS® Magnetic Resonance Imaging. In: ACR BI-RADS® Atlas, Breast Imaging Reporting and Data System. 5th edition. Reston, VA, American College of Radiology; 2013. p 125-143.

# Supplementary Figures


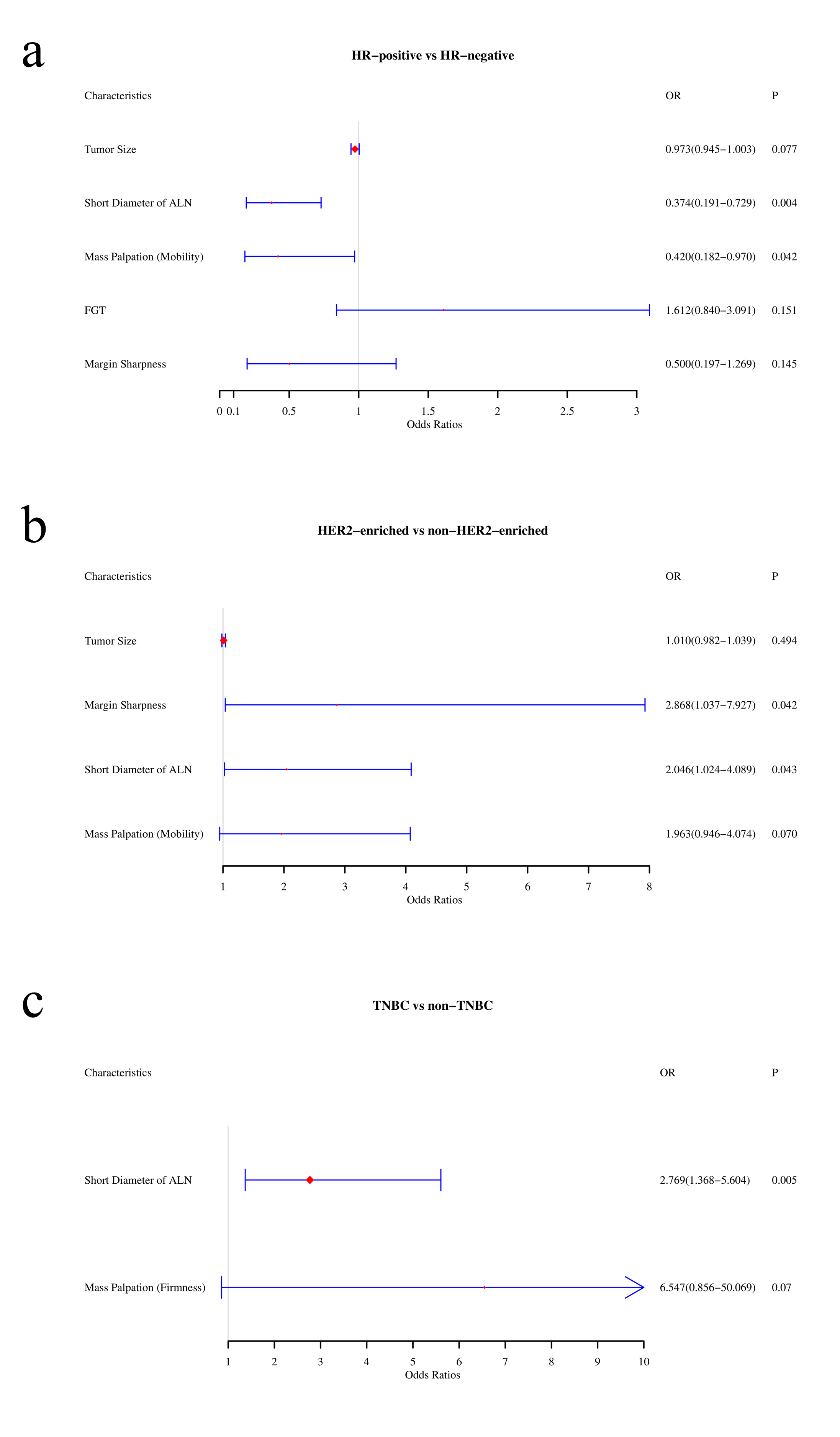


**Supplementary Figure S1.** Forest plots of clinical-radiological characteristics in Tasks 1-3. Based on the multivariate binary logistic regression, the odds ratios were calculated and independent clinical-radiological predictors were selected by P-value (P < 0.05). (a) Forest plot of Task 1 (HR-positive vs HR-negative). (b) Forest plot of Task 2 (HER2-enriched vs non-HER2-enriched). (c) Forest plot of Task 3 (TNBC vs non-TNBC).


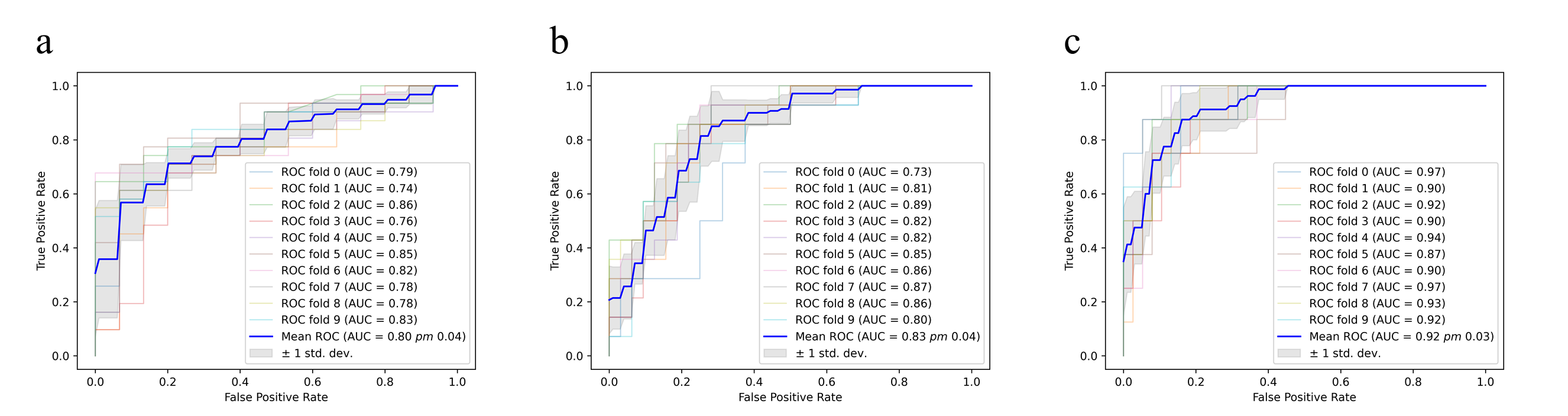


**Supplementary Figure S2.** 10-fold cross-validation of the CCRMs in task 1-3. (a) 10-fold cross-validation of the CCRMs in task 1. (b) 10-fold cross-validation of the CCRMs in task 2. (c) 10-fold cross-validation of the CCRMs in task 3. The blue line represents the average result of ten cross-validations. The grey shaded area is the corresponding standard deviation.


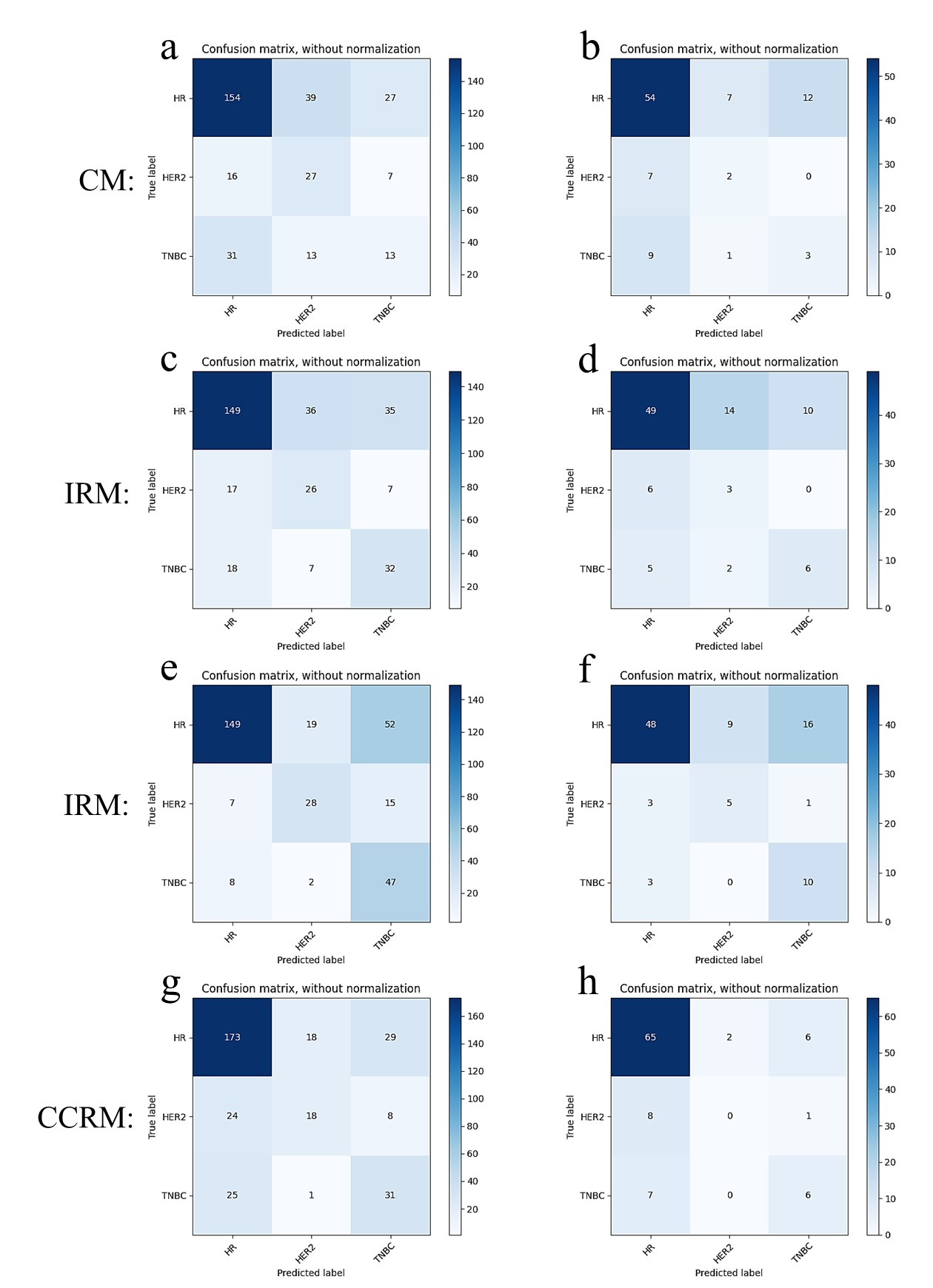


**Supplementary Figure S3.** Confusion matrix of CM, IRM, PRM and CCRM in task 4. (a) Confusion matrix of CM in training cohorts. (b) Confusion matrix of CM in test cohorts. (c) Confusion matrix of IRM in training cohorts. (d) Confusion matrix of IRM in test cohorts. (e) Confusion matrix of PRM in training cohorts. (f) Confusion matrix of PRM in test cohorts. (g) Confusion matrix of CCRM in training cohorts. (h) Confusion matrix of CCRM in test cohorts. CM: clinical-radiological model. IRM: intratumoral radiomics model. PRM: peritumoral radiomics model. CCRM: combined clinical-radiological and radiomics model.

# Supplementary Tables

**Table S1. Classification of Molecular Subtypes**

| Molecular Subtype | ER |  | PR | HER2 |
| --- | --- | --- | --- | --- |
| HR-positive | positive | and/or | positive | positive or negative |
| HER2-enriched | negative |  | negative | positive |
| TNBC | negative |  | negative | negative |

Abbreviations: ER Estrogen receptor; HER2 Human epidermal growth factor receptor 2; HR Hormone receptor; PR Progesterone receptor; TNBC Triple-negative breast cancer.

**Table S2. Breast MRI sequences and acquisition parameters in two centers**

| Hospital | Coil | Contrast agent | Sequence | TR (ms) | TE (ms) | FOV (mm) | Matrix | Slice thickness (mm) | Flip angle | b value |
| --- | --- | --- | --- | --- | --- | --- | --- | --- | --- | --- |
| Center 1 | 7-element SENSE breast coil | GD-DTPA (Magnevist), Beilu Pharmaceutical, Beijing, China | T1WI | 5.7 | 2.4 | 340×340 | 234×225 | 4.5 | 90 | - |
|  |  |  | T2WI | 5000 | 60 | 337×337 | 336×303 | 4.5 | 90 | - |
|  |  |  | DWI | 3000 | 59 | 406×406 | 188×186 | 4.5 | 90 | 800 |
|  |  |  | DCE | 4.6 | 2.2 | 355×355 | 324×271 | 2 | 10 | - |
| Center 2 | 4-channel breast-array coil | GD-DTPA (Magnevist), Beilu Pharmaceutical, Beijing, China | T1WI | 4.8 | 2.3 | 350×350 | 320×244 | 4 | 90 | - |
|  |  |  | T2WI | 3360 | 38.6 | 350×350 | 320×160 | 4 | 90 | - |
|  |  |  | DWI | 4500 | 73.6 | 350×350 | 128×128 | 4 | 90 | 800 |
|  |  |  | DCE | 4.9 | 2.2 | 380×380 | 380×288 | 3.6 | 15 | - |

For the DCE sequence, the six phases image with 60s per phase in Center 1 and seven phases with 70s per phase in Center 2 were acquired after the end of high-pressure syringe injection. Abbreviations: FOV field of view; TE echo time; TR repetition time.

**Table S3. Clinical-radiological predictors in task 1-4**

| Tasks | | Characteristics | P | OR (95% CI) |
| --- | --- | --- | --- | --- |
| Task 1 | | Short diameter of ALN | < 0.001* | 0.264 (0.146-0.479) |
|  |  | Mass palpation (Mobility) | 0.018* | 0.376 (0.168-0.843) |
| Task 2 | | Margin sharpness | 0.025* | 3.146 (1.158-8.547) |
|  |  | Short diameter of ALN | 0.004* | 2.374 (1.312-4.298) |
| Task 3 | | Short diameter of ALN | 0.003* | 2.886 (1.436-5.800) |
| Task 4 | HR-positive vs HER2-enriched | Tumor size | < 0.001* | 1.061 (1.035-1.088) |
|  | HR-positive vs TNBC | Tumor size | 0.048* | 1.027 (1.000-1.054) |
|  | HER2-enriched vs TNBC | Tumor size | 0.029* | 1.033 (1.003-1.064) |

Clinical-radiological predictors were used to developed the CMs in each task. P is derived from multivariate logistic regression analyses, and P* < 0.05 is considered statistically significant. Task 1: HR-positive vs HR-negative; Task 2: HER2-enriched vs non-HER2-enriched; Task 3: TNBC vs non-TNBC; Task 4: HR-positive vs HER2-enriched vs TNBC. Abbreviations: ALN Axillary lymph node; CI Confidence interval; HER2 Epidermal growth factor receptor 2; HR hormone receptor; TNBC Triple-negative breast cancer; OR odds ratio.

**Table S4. Intratumoral and peritumoral radiomics features in task 1-4.**

| Task |  | Intratumoral | | |  | Peritumoral | | |
| --- | --- | --- | --- | --- | --- | --- | --- | --- |
|  |  | Image Filtering | Feature Type | Features |  | Image Filtering | Feature Type | Features |
| Task 1 |  | LOG | First order | Maximum |  | Exponential | Glcm | Imc1 |
|  |  | Original | Shape | Flatness |  | Original | Shape | Major Axis Length |
|  |  | Square root | Gldm | Small Dependence Low Gray Level Emphasis |  | Original | Shape | Maximum2DDiameterRow |
|  |  | Wavelet | Glszm | Zone Entropy |  | Original | Shape | Sphericity |
|  |  | Wavelet | Glcm | Imc1 |  | Wavelet | First order | Total Energy |
|  |  | Wavelet | Ngtdm | Busyness |  | Wavelet | Glcm | Imc2 |
|  |  | - | - | - |  | Wavelet | Gldm | Dependence NonUniformity |
|  |  | - | - | - |  | Wavelet | Glszm | Gray Level NonUniformity Normalized |
| Task 2 |  | Exponential | Glcm | Cluster Prominence |  | Original | Glrlm | Run Entropy |
|  |  | Exponential | Glcm | Imc1 |  | Original | Shape | Elongation |
|  |  | LOG | Gldm | Large Dependence High Gray Level Emphasis |  | Square root | Glrlm | Short Run High Gray Level Emphasis |
|  |  | Original | Shape | Flatness |  | Wavelet | Glszm | Gray Level NonUniformity |
|  |  | Square | Glrlm | Gray Level Variance |  | Wavelet | First order | Kurtosis |
|  |  | Square root | Gldm | Dependence Variance |  | Wavelet | Glcm | Correlation |
|  |  | Wavelet | Glrlm | Short Run Emphasis |  | Wavelet | First order | Mean |
|  |  | Wavelet | First order | Mean |  | Wavelet | First order | Uniformity |
|  |  | Wavelet | Glcm | Correlation |  | Wavelet | Glcm | Imc1 |
|  |  | Wavelet | Glszm | Small Area Emphasis |  | Wavelet | Glcm | Joint Entropy |
|  |  | Wavelet | First order | Kurtosis |  | Wavelet | Gldm | Dependence NonUniformity |
|  |  | Wavelet | Glrlm | Run Entropy |  | - | - | - |
| Task 3 |  | LOG | First order | 90Percentile |  | Exponential | Glcm | Imc1 |
|  |  | LOG | First order | Skewness |  | LOG | First order | Median |
|  |  | Square root | Gldm | Small Dependence Low Gray Level Emphasis |  | Wavelet | First orde | Median |
|  |  | Wavelet | Glszm | Zone Entropy |  | Wavelet | First order | Mean |
|  |  | Wavelet | First order | 90Percentile |  | Wavelet | Glszm | Gray Level NonUniformity Normalized |
|  |  | - | - | - |  | Wavelet | Ngtdm | Coarseness |
|  |  | - | - | - |  | Wavelet | Glcm | Imc1 |
|  |  | - | - | - |  | Wavelet | Glcm | MCC |
| Task 4 |  | LOG | First order | 90Percentile |  | Exponential | Glcm | Imc1 |
|  |  | LOG | First order | Skewness |  | LOG | First order | Energy |
|  |  | LOG | Glszm | Gray Level NonUniformity |  | Original | Shape | Elongation |
|  |  | Logarithm | Gldm | Small Dependence Low Gray Level Emphasis |  | Original | Shape | Least Axis Length |
|  |  | Original | Shape | Flatness |  | Original | Shape | Maximum2DDiameterRow |
|  |  | Original | Shape | Major Axis Length |  | Original | Shape | Maximum2DDiameterSlice |
|  |  | Original | Shape | Maximum2DDiameterRow |  | Square root | Ngtdm | Coarseness |
|  |  | Original | Shape | Maximum2DDiameterSlice |  | Wavelet | Glszm | Large Area High Gray Level Emphasis |
|  |  | Wavelet | Gldm | Small Dependence Low Gray Level Emphasis |  | Wavelet | Gldm | Dependence NonUniformity |
|  |  | Wavelet | First order | Energy |  | Wavelet | Ngtdm | Strength |
|  |  | Wavelet | Glszm | Gray Level NonUniformity |  | - | - | - |

Task 1: HR-positive vs HR-negative. Task 2: HER2-enriched vs non-HER2-enriched. Task 3: TNBC vs non-TNBC. Task 4: HR-positive vs HER2-enriched vs TNBC. First order: First-order Statistic Features. Glcm: Gray Level Co-occurrence Matrix (GLCM) Features. Glrlm: Gray Level Run Length Matrix (GLRLM) Features. Glszm: Gray Level Size Zone Matrix (GLSZM) Features. Ngtdm: Neighbouring Gray Tone Difference Matrix (NGTDM) Features. Gldm: Gray Level Dependence Matrix (GLDM) Features.

**Table S5. Different Models Performance in task 4.**

| Model | Classify | Training cohort | | | |  | Test cohort | | | |
| --- | --- | --- | --- | --- | --- | --- | --- | --- | --- | --- |
|  |  | Accuracy | Precision | Recall | F1-Score |  | Accuracy | Precision | Recall | F1-Score |
| CM | HR | 0.593 | 0.77 | 0.7 | 0.73 |  | 0.621 | 0.77 | 0.74 | 0.76 |
|  | HER2 |  | 0.34 | 0.54 | 0.42 |  |  | 0.2 | 0.22 | 0.21 |
|  | TNBC |  | 0.28 | 0.23 | 0.25 |  |  | 0.2 | 0.23 | 0.21 |
| IRM | HR | 0.633 | 0.81 | 0.68 | 0.74 |  | 0.611 | 0.82 | 0.67 | 0.74 |
|  | HER2 |  | 0.38 | 0.52 | 0.44 |  |  | 0.16 | 0.33 | 0.21 |
|  | TNBC |  | 0.43 | 0.56 | 0.49 |  |  | 0.38 | 0.46 | 0.41 |
| PRM | HR | 0.685 | 0.91 | 0.68 | 0.78 |  | 0.663 | 0.89 | 0.66 | 0.76 |
|  | HER2 |  | 0.57 | 0.56 | 0.57 |  |  | 0.36 | 0.56 | 0.43 |
|  | TNBC |  | 0.41 | 0.82 | 0.55 |  |  | 0.37 | 0.77 | 0.5 |
| CIPRM | HR | 0.697 | 0.91 | 0.7 | 0.79 |  | 0.663 | 0.89 | 0.66 | 0.76 |
|  | HER2 |  | 0.62 | 0.56 | 0.59 |  |  | 0.36 | 0.56 | 0.43 |
|  | TNBC |  | 0.41 | 0.82 | 0.55 |  |  | 0.37 | 0.77 | 0.5 |
| CCRM | HR | 0.678 | 0.78 | 0.79 | 0.78 |  | 0.747 | 0.81 | 0.89 | 0.85 |
|  | HER2 |  | 0.49 | 0.36 | 0.41 |  |  | 0 | 0 | 0 |
|  | TNBC |  | 0.46 | 0.54 | 0.5 |  |  | 0.46 | 0.46 | 0.46 |

Abbreviations: CM Clinical-radiological models; CIPRM Combined intra- and peri-tumoral radiomics model; CCRM Combined clinical-radiological and radiomics models; HER2 Human epidermal growth factor receptor 2; HR Hormone receptor; IRM Intratumoral radiomics model; PRM Peritumoral radiomics model; TNBC Triple-negative breast cancer.
